# Partial-body cryostimulation procured performance and perceptual improvements in amateur middle-distance runners

**DOI:** 10.1371/journal.pone.0288700

**Published:** 2023-11-22

**Authors:** Massimo De Nardi, Luca Filipas, Carlo Facheris, Stefano Righetti, Marco Tengattini, Emanuela Faelli, Ambra Bisio, Gabriele Gallo, Antonio La Torre, Piero Ruggeri, Roberto Codella

**Affiliations:** 1 Krioplanet Ltd, Treviglio, Bergamo, Italy; 2 Department of Experimental Medicine, Università degli Studi di Genova, Genoa, Italy; 3 Department of Biomedical Sciences for Health, Università degli Studi di Milano, Milan, Italy; 4 Department of Endocrinology, Nutrition and Metabolic Diseases, IRCCS MultiMedica, Milano, Italy; 5 Italian Athletics Federation, Rome, Italy; 6 Interventional Cardiology Department, San Gerardo Hospital, Monza, Italy; 7 Centro Polifunzionale di Scienze Motorie, Università degli Studi di Genova, Genoa, Italy; 8 IRCCS Istituto Ortopedico Galeazzi, Milan, Italy; University of Ljubljana, Medical faculty, SLOVENIA

## Abstract

The purpose of this study was to investigate the effects of partial-body cryostimulation on middle-distance runners before two 3000-m tests at the speed of the first and second ventilatory threshold, and before a time to exhaustion test at 110% of the maximal aerobic speed. Twelve amateur runners (age: 46 ± 9 years; VO_2max_: 51.7 ± 4.9 ml·kg^-1^·min^-1^) completed six running testing sessions in a randomized counterbalanced cross-over fashion: three of them were preceded by a partial-body cryostimulation and the other three by a control condition. The testing sessions consisted of: 1) a 3000-m continuous running test at the speed of the first ventilatory threshold; 2) a 3000-m continuous running test at the speed of the second ventilatory threshold; 3) a time to exhaustion test at 110% of the maximal aerobic speed. Heart rate, ratings of perceived exertion and visual analogue scale relative to muscle pain were recorded throughout the tests. Total quality recovery was evaluated 24–48 h after the end of each test. Distance to exhaustion was higher after partial-body cryostimulation than control condition (p = 0.018; partial-body cryostimulation: 988 ± 332 m, control: 893 ± 311 m). There were differences in the ratings of perceived exertion during each split of the 3000-m continuous running test at the speed of the second ventilatory threshold (p = 0.001). Partial-body cryostimulation can be positively considered to enhance middle-distance running performance and reduce perception of effort in amateur runners.

## Introduction

In high-performance sport, coaches and sports scientists strive to identify novel interventions to enhance performance maximizing the physical, psychological and behavioral state before a competition [1, 2]. Prior to performance, several routines have been pursued with the aim of enhancing oxygen uptake, cardiac output, blood flow to skeletal muscle, neuromuscular activation and mental readiness [3]. Running economy is certainly another typical target.

Pre-training typically involves a combination of passive and active elements which can enhance exercise performance [4]. Active warm-ups are used to increase body temperature leading to a higher muscle metabolism, enhanced oxygen uptake and subsequent cardiac output [5], while passive warm up has the same goal but is mainly focused on various psychological techniques or cooling/heating interventions [6]. Preparation through passive modalities is becoming increasingly common for athlete management and performance enhancement [7]. Although every team, staff, strength and conditioning coach, have individualized methods and preferences for their athletes/players, cutting-edge methodologies and techniques are evolving and being available at large scale [8].

Cryotherapy is a relatively new clinical intervention used in different medical treatments to alleviate pain derived from inflammatory conditions [9]. Given the extremely cold air temperatures of –110°C or below, reached in short (1–4 min) exposures, cryostimulation can elicit strong physiological responses in users [10], ranging from muscular soreness relief up to a modest immunomodulatory effect [11].

The utilization of cryostimulation techniques before physical efforts is also known as precooling [12]. Usually, precooling aims to a rapid removal of heat of the body prior to exercising in warm environments and to prevent the side effects of heat-stress-induced fatigue [13]. Reducing body temperature before commencing an effort has been found to be successful in improving endurance performance both in hot and thermoneutral environments [14, 15]. Higher precooling effects were also observed in prolonged efforts [16], ambient temperature and for aerobic capacity [17]. Improvements in endurance performance following precooling interventions were achieved both with external (cold air exposure, water immersion, exposure to ice, iced garments, iced towels, etc.), internal modalities (beverage ingestion, ice ingestion) or combining two or more practical precooling methods [12, 17]. Cooling strategies are logistically challenging, therefore it is important to analyze the effectiveness of practical precooling in competition or field setting [12].

In particular, the new transportable models of cryo-saunas have increased the diffusion of partial-body cryostimulation (PBC) treatments before races and training [18], which have been demonstrated to lower core and skin temperature for several hours [19]. Exposures to very-low temperatures (approximately at -120°C) could have positive impacts on core body temperature, cardiovascular and autonomic functions [20], and perception of effort [21]. Nevertheless, the disparities in the literature among various precooling methods, exercise efforts and thermometry protocols remain substantial. This breath of knowledge could be scoped by athletes, coaches and their extended entourage to tailor the maximizing-performance routine.

In this study, the effects of PBC on running performance were evaluated with two 3000-m tests at the speed of the first and second ventilatory threshold, and before a time to exhaustion test at 110% of the maximal aerobic speed (MAS). We aimed to test if PBC could be applied as a prior to competition performance enhancement methodology in middle-distance events. Our hypothesis was that running performance would have been improved by cryo-exposures, along with lower value of perceptual fatigue.

## Materials & methods

### Experimental approach to the problem

**Fig 1:**
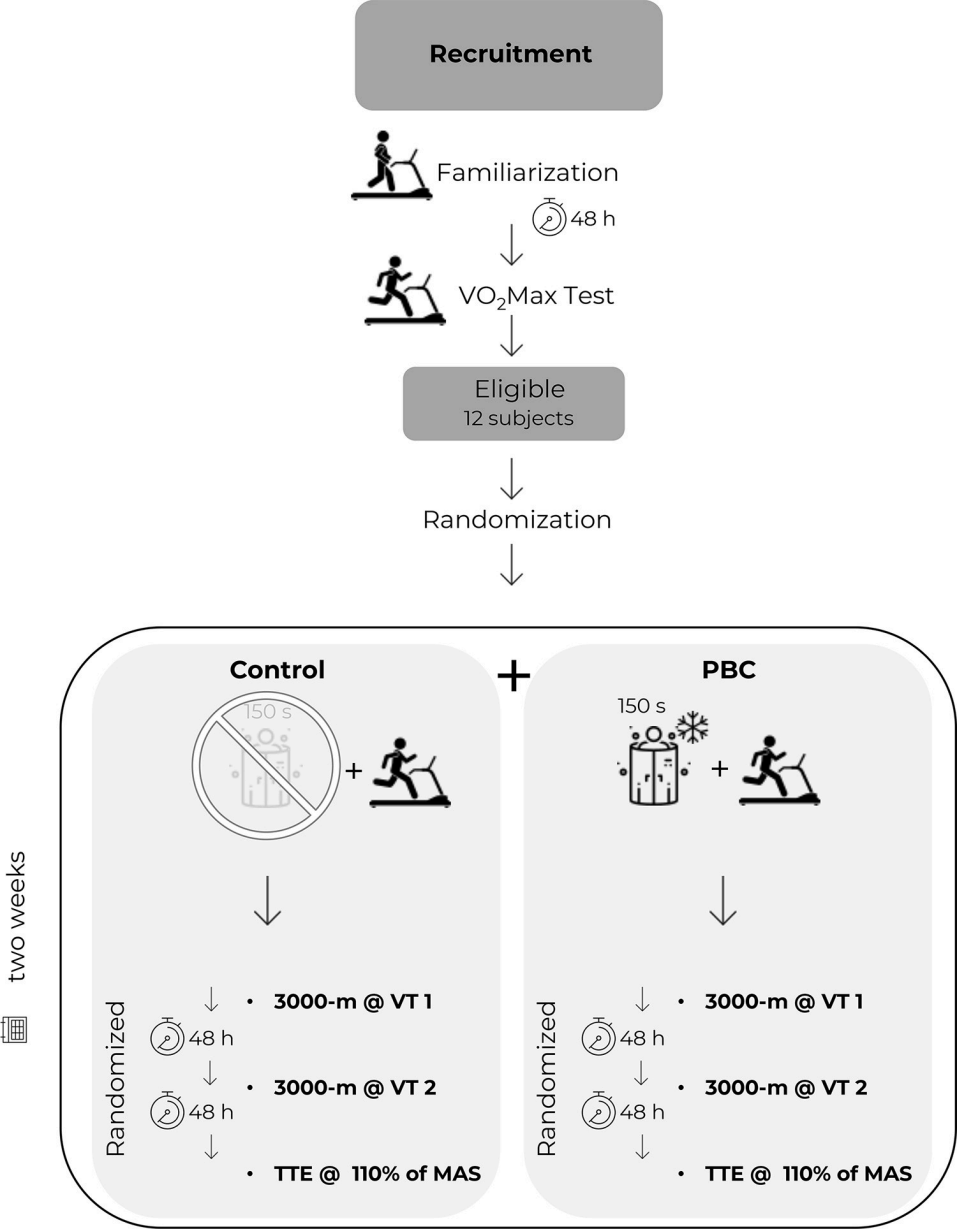
Flow-chart of the study.

A randomized counterbalanced cross-over design was used for the experimental component of the present study. The order of the experimental treatments (PBC; control) and testing sessions were randomly allocated based on balanced permutations generated by a computer program (www.randomization.com). A flow-chart of the protocol is offered in Fig 1.

### Subjects

To determine an *a priori* sample-size (software package, G * Power 3.1.9.2), the following input parameters were selected as per an F test for ANOVA-repeated measures-within factors analysis: a statistical power (1-β) of 0.8, a significance α level of 0.05, an effect size *f* of 0.38 (which corresponds to a η^2^_p_ = 0.13), 1 group, 6 measurements, 0.5 as correlation among repeated measurements. As output parameters, an actual power of 0.83, a critical F of 2.45 were obtained. Therefore, eight subjects would have been sufficient to assess the sought effects. However, to face a possible drop-out of one third of the subjects, thirteen male amateur runners were recruited for this study. Participants were evaluated by a medical doctor who ascertained their competitive suitability and excluded any contraindication to systemic cryostimulation. Eligibility criteria were as follows: being free from any known medical diseases, medication, injuries, color vision deficiencies. The study design and procedures were approved by the local Ethics Committee and followed the ethical principles for medical research involving human subjects set by the World Medical Association Declaration of Helsinki. After ethical approval, written informed consent and medical declaration were obtained from the participants in line with the procedures set by the local Institution’s Research Ethics Committee. Subjects were informed of the procedures and potential risks involved, although these were minimal as already described [22]. Participants were also informed that they could withdraw from the study at any time, for any reason.

### Procedures

Participants performed eight testing sessions on eight different occasions, in a period no longer than three weeks between the first and last visit (with at least 48 h between two visits). Visits were carried in a private laboratory. All the experimental procedures were performed in an isolated and air-conditioned room, at the constant temperature of 21 ± 1°C and at a relative humidity of 40–50%. Before each visit, participants were instructed to sleep for at least 8 h, refrain from the consumption of alcohol and caffeine, and avoid any vigorous exercise for the 24-h preceding the testing sessions. Each participant carried out the visits individually and at the same time of day (within 1 h period, between 7:30 and 10:30).

During visit 1, participants’ body weight and height were measured. Afterwards, they familiarized with the procedures employed for the testing sessions, i.e. running on the treadmill used for the study (Excite Treadmill, Technogym, Cesena, Italy) for at least 20 min wearing a portable gas exchange system in breath-by-breath mode (Cosmed K5, Cosmed, Rome, Italy). Treadmill speed was validated for each stage using an odometer (Stanley, Milano, Italy) to confirm that it was the one declared by the manufacturer. During visit 2, participants completed an incremental exercise test to determine their maximal oxygen uptake (VO_2max_). The test started with a standardized warm-up consisting in 10 min of running at a constant speed of 10 km·h^-1^, followed by 5 min of mobility drills. At the end of the mobility exercises, participants completed 10 min of passive recovery and, after wearing the mask, 3 min in a standing position on the treadmill for the acquisition of basal values. After the warm-up, the incremental test started at a speed equal to 10 km·h^-1^, with an increase of 0.1 km·h^-1^ every 12 s until exhaustion. Alveolar gas exchanges were measured breath by breath in the mouth, eliminating any values outside the pre-established range, using a specific software (OMNIA, Cosmed, Rome, Italy). Maximum oxygen uptake was calculated as the 30 s mean oxygen uptake once the plateau was reached. Runner’s VO_2max_ was reached when at least three of the following criteria were fulfilled: i) a steady state of VO_2_ (change in VO_2_ at VO_2max_ ≤ 150 mL·min^-1^), ii) final respiratory-exchange ratio (RER) exceeded 1.1, iii) visible exhaustion, iv) a HR at the end of exercise within the 10 bpm of the predicted maximum, and v) a lactate concentration at the end of exercise higher than 8 mmol·L^-1^ [23]. Blood samples were obtained immediately after exhaustion, in a single measurement, through the ear lobe, and were analyzed for whole blood lactate using a portable lactate analyzer (Lactate Pro, Arcray Inc, Kyoto, Japan), reported to have good reliability and accuracy [24]. First and second ventilatory thresholds were detected through the ventilatory equivalents method [25] and the speeds associated were calculated. In addition, researchers calculated the maximal aerobic speed (MAS) as the lowest running speed at which VO_2max_ occurred.

After the first two preliminary visits, participants carried out randomly the three testing sessions, preceded either by a PBC or a control condition (six sessions in total, all randomized, Fig 1). The testing sessions consisted of a 3000-m continuous running test at the speed of the first ventilatory threshold, a 3000-m continuous running test at the speed of the second ventilatory threshold and a time to exhaustion test at 110% of the MAS. The order of the tests and the interventions were randomly counterbalanced. The tests were preceded by the same warm-up routine described for the incremental ramp test. The warm-up started within two minutes by the end of the PBC or control condition. The tests at the first and second ventilatory thresholds were performed over a distance of 3000 m to give enough time to the physiological parameters to reach a steady-state.

### Experimental treatments

Before each testing session, participants underwent either a PBC session or a control session. During the PBC duty, participants completed the cryo-session (150 s) in a cryo-cabin (Space Cabin, Criomed Ltd, Kherson, Ukraine). Temperature was set between -130 and -170°C as recommended [26]. Participants were instructed to turn around continuously (standing rotations) in the cabin for the 150-second session. The control condition required to perform similar movement (standing rotations) for the same duration in a thermo neutral environment (21 ± 1°C). Immediately after the cryo-exposure or control task, the running tests were performed. Due to the nature of the cryostimulation, participants were not blinded to their treatments. However, the research team was blinded to the treatment because specific personnel oversaw the treatments in a separated room.

### Physiological and psychological measures

Heart rate (HR) was recorded, and averaged for the duration of the tests, using a HR monitor fitted with a chest strap. VO_2_ was recorded using portable gas exchange system in breath-by-breath mode (Cosmed K5, Cosmed, Rome, Italy) and data were averaged using the specific software (OMNIA, Cosmed, Rome, Italy). During the 3000-m continuous running tests and the 110% of the MAS test, VO_2_ were reported as the average of the last 30 s of the tests. Rating of perceived exertion (RPE) was registered during the final 10 s of 1000- and 2000-m splits, at the end of the 3000-m continuous running tests, and at the end of the time to exhaustion test. RPE was measured with the 11-point CR10 scale developed by Borg [27]. Participants were familiar with the scale as it had been employed during their daily training sessions for at least six months ahead the tests. Participants recorded their subjective sensation of muscle pain by using a visual analogue scale (VAS), before and after each test. Participants marked their response on a 100-mm line anchored by 0 (no muscle pain at all) and 100 (maximum muscle pain) [28]. The total quality recovery (TQR) scale [29] was used to monitor recovery. After 24 and 48 hours from the end of each test, participants answered the question “How do you feel about your recovery?” using the TQR scale, in which answers are rated from 6 to 20.

### Statistical analyses

All data are presented as mean ± standard deviation. Assumptions of statistical tests such as normal distribution (Shapiro-Wilk test with visual inspection) and sphericity of data (Mauchly’s test) were checked as appropriate. Greenhouse-Geisser correction to the degrees of freedom was applied when violation to sphericity was present. Two-way repeated measured ANOVA was used to determine the treatment factor (2 levels, PBC and Control), time factor (3 levels, at 1000 m, 2000 m, 3000 m splits), and interaction on RPE during the 3000-m tests. Significant main effects and interactions were interpreted through pairwise comparisons with Bonferroni correction. Paired sample t tests were used to compare the other variables between the PBC and control conditions. Significance was set at 0.05 (two-tailed) for all analyses. Effect sizes for repeated measure ANOVA are reported as partial eta squared (η^2^_p_), using the small (< 0.13), medium (0.13–0.25) and large (> 0.25) interpretation for effect size [30], while effect sizes for pairwise comparison were calculated using Cohen’s *d* and considered to be either trivial (effect size: < 0.20), small (0.21–0.60), moderate (0.61–1.20), large (1.21–2.00), or very large (> 2.00) [31]. Data analysis was conducted using the Statistical Package for the Social Sciences, version 25 (SPSS Inc., Chicago, IL, USA).

## Results

Participants’ baseline characteristics and data derived from the incremental ramp test are reported in Table 1. One participant was classified as outlier based on his maximum oxygen uptake (VO_2max_) (more than two standard deviation from the mean of the sample) and excluded from the analysis. Therefore, twelve subjects were included in the study procedures (age: 46 ± 9 years; height: 1.75 ± 0.05 m; mass: 72 ± 8 kg).

**Table 1:** Participants’ characteristics at baseline (mean ± SD).

VO_2max_ (mL·kg^-1^·min^-1^)	51.7 ± 4.9
HR_max_, incremental test (bpm)	181 ± 16
v_VT1_ (km·h^-1^)	11.8 ± 0.9
v_VT2_ (km·h^-1^)	13.4 ± 0.9
MAS (km·h^-1^)	14.7 ± 1.1
VO_2VT1_ (mL·kg^-1^·min^-1^)	45.2 ± 5.3
VO_2VT2_ (mL·kg^-1^·min^-1^)	49.8 ± 5.3
HR_VT1_ (bpm)	156 ± 18
HR_VT2_ (bpm)	167 ± 17

VO_2max_: maximum oxygen uptake; HR_max_: maximum heart rate; v_VT1_: velocity at first ventilatory threshold; v_VT2_: velocity at second ventilatory threshold; MAS: maximum aerobic speed; VO_2VT1_: oxygen uptake at first ventilatory threshold; VO_2VT2_: oxygen uptake at second ventilatory threshold; HR_VT1_: heart rate at first ventilatory threshold; HR_VT2_: heart rate at second ventilatory threshold.

### Performance outcomes

Table 2 shows the performance outcomes derived from the 3000-m continuous running test at the speed of the first and second ventilatory threshold and the time to exhaustion test at 110% of the MAS. Running distance (p = 0.018, *d* = 0.30) and time (p = 0.020, *d* = 0.31) during the time to exhaustion test were higher after PBC than control condition. No differences were found among the other parameters.

**Table 2:** Performance outcomes from the three running tests in partial-body cryotherapy (PBC) and control conditions (mean ± SD).

**3000-m test at VT1**	**PBC**	**Control**	**p**	**Cohen’s *d***
Time (s)	910 ± 77	919 ± 70	0.237	0.13
VO_2_ (mL·kg^-1^·min^-1^)	46.4 ± 5.5	47.1 ± 6.0	0.731	0.12
HR (bpm)	159 ± 17	159 ± 18	0.478	0.04
RER (VCO_2_·VO_2_^-1^)	0.84 ± 0.06	0.85 ± 0.05	0.750	0.13
**3000-m test at VT2**				
Time (s)	693 ± 217	711 ± 152	0.491	0.09
VO_2_ (mL·kg^-1^·min^-1^)	48.8 ± 5.8	48.1 ± 5.2	0.502	0.13
HR (bpm)	168 ± 15	167 ± 17	0.625	0.05
RER (VCO_2_·VO_2_^-1^)	0.92 ± 0.08	0.92 ± 0.09	0.817	0.06
**TTE at 110% of MAS**				
Distance (m)	988 ± 332	893 ± 311	0.018 *	0.30
Time (s)	222 ± 73	201 ± 67	0.020 *	0.31
VO_2_ (mL·kg^-1^·min^-1^)	49.8 ± 6.1	46.7 ± 6.2	0.233	0.49
HR (bpm)	170 ± 13	167 ± 13	0.227	0.21
RER (VCO_2_·VO_2_^-1^)	1.13 ± 0.11	1.13 ± 0.12	0.781	0.07

VT1: first ventilatory threshold; VT2: second ventilatory threshold; TTE: time to exhaustion; MAS: maximum aerobic speed; VO_2_: oxygen uptake; HR: heart rate; RER: respiratory exchange ratio.

* Significant difference between the conditions (p < 0.05).

### Psychological outcomes

Table 3 shows the pairwise comparison of the psychological outcomes derived during the 3000-m continuous running test at the speed of the first and second ventilatory threshold and the time to exhaustion test at 110% of the MAS. There was a significant condition x time interaction in the RPE during the 3000-m continuous running test at the speed of the second ventilatory threshold (F _(2,18)_ = 15.716, p = 0.001, η^2^_p_ = 0.43). Pairwise comparison revealed significant differences for RPE in 1000-m (p = 0.010, *d* = 0.54), 2000-m (p = 0.030, *d* = 0.65) and 3000-m (p = 0.008, *d* = 0.56) splits. No differences were found among the other parameters.

**Table 3:** Psychological outcomes from the three running tests in partial-body cryotherapy (PBC) and control conditions (mean ± SD).

**3000-m test at VT1**	**PBC**	**Control**	**p**	**Cohen’s *d***
VAS pre (mm)	11 ± 14	18 ± 16	0.151	0.44
VAS post (mm)	15 ± 18	17 ± 17	0.638	0.10
1000-m RPE	2.6 ± 1.2	2.3 ± 0.9	0.410	0.24
2000-m RPE	3.2 ± 1.5	3.0 ± 1.2	0.305	0.13
3000-m RPE	3.3 ± 1.9	3.3 ± 1.9	1.000	0.00
24-h TQR	17.5 ± 2.3	17.5 ± 2.6	0.915	0.02
48-h TQR	18.6 ± 2.1	18.1 ± 2.3	0.053	0.35
**3000-m test at VT2**				
VAS pre (mm)	13 ± 11	11 ± 12	0.504	0.15
VAS post (mm)	13 ± 11	18 ± 17	0.141	0.41
1000-m RPE	3.2 ± 1.3	3.9 ± 1.5	0.010 *	0.54
2000-m RPE	4.3 ± 1.7	5.5 ± 2.1	0.030 *	0.65
3000-m RPE	5.3 ± 1.8	6.6 ± 2.7	0.008 *	0.56
24-h TQR	17.9 ± 2.1	16.3 ± 3.3	0.091	0.57
48-h TQR	18.2 ± 2.4	17.2 ± 2.4	0.090	0.44
**TTE at 110% of MAS**				
VAS pre (mm)	8 ± 11	10 ± 14	0.748	0.14
VAS post (mm)	15 ± 14	23 ± 28	0.279	0.36
RPE at exhaustion	8.2 ± 2.0	8.3 ± 2.0	0.928	0.02
24-h TQR	17.2 ± 2.0	17.1 ± 2.1	0.903	0.04
48-h TQR	17.8 ± 1.8	17.3 ± 2.7	0.324	0.22

VT1: first ventilatory threshold; VT2: second ventilatory threshold; TTE: time to exhaustion; MAS: maximum aerobic speed; VAS: visual analogue scale; RPE: rating of perceived exertion; TQR: total quality recovery.

* Significant difference between the conditions (p < 0.05).

## Discussion

The main finding of this study was that a PBC session increased running time to exhaustion, showing an improved performance at 110% of MAS after a single PBC session compared to a control condition. Further, RPE was significantly reduced after the PBC in each split of the 3000-m continuous running test at the second ventilatory threshold, suggesting that both central and peripheral mechanisms could be affected by PBC. Partially unexpected, no other differences were found in the two different conditions for other performance or psychological measures.

This study showed a potential implication in adopting this relatively new technique before running performance in a moderate-temperature condition. To date, an abundance of evidence demonstrated the favorable use of PBC as a recovery modality after high intensity exercise for athletes. Researchers have identified how PBC can improve recovery post-exercise by maximizing anti-inflammatory and decreasing pro-inflammatory actions [9]. Conversely, to the best of our knowledge, the effects of PBC before an exercise are mostly uncertain. Prior investigations have documented the effectiveness of precooling using PBC in ameliorating flexibility without losing the trunk position sense proprioception [32]. In addition, numerous studies showed the positive influence of several precooling strategies in hot environments on endurance performance, while no studies have evaluated the effects of PBC in thermoneutral conditions [14]. Our results indicated PBC as a method capable to induce improvements in middle-distance running performance, probably mediated by a lower RPE for the same external workload. This is only a hypothesis as in the present study the improvement in performance and the reduction of RPE occurred not in combination but during different tests. This result is possibly due to the timing of the RPE evaluation, i.e. at the end of the time to exhaustion test, and at each 1000-m split in the 3000-m time trials. Therefore, the ceiling effect of the RPE in an exercise-to-exhaustion probably determined the lack of significance in the TTE at 110% of the MAS [27]. This result is not an unicum in the body of literature as a similar one was found in a submaximal exercise in elite synchronized swimmers [33]. Klimek and colleagues have also shown an improvement in anaerobic capacity after 10 whole-body cryostimulation sessions, principally explained by metabolic changes (i.e. increased activity of anaerobic glycolytic enzymes) and a greater tolerance to pain, highlighted by an increase in blood lactate concentration [34].

Reduction in pain could be easily linked to a decrease in perception of effort, a master regulator of performance in endurance exercises [35]. Of note, perception of effort is determinant in a TTE test, as it has been shown that maximum values of perception of effort lead to an early stop to exercise, despite athletes could continue both muscularly and metabolically [36].

Physiologically, the reduction of perception of effort could be explained by the modification of peripheral afferent signals generated by PBC, that could play a crucial role in changing the perception of effort during exercise at low- and high-intensity [37]. In fact, together with neural drive from the motor cortex area, previous studies showed that RPE also is influenced by afferent feedback from the periphery for the prediction of the endpoint of the exercise bout [37, 38].

No other changes were observed in the other physiological and psychological parameters. Our results are in line with previous studies that have shown no change in performance, recovery, soreness perception parameters [39, 40], and with strategies that altered the perception of effort [41, 42]. Moreover, the reduction of RPE at the same timepoints in the 3000-m tests implies that the RPE:VO_2_ and RPE:HR ratios are reduced in both tests in the PBC condition compared to the control one. This reduction denotes an indirect change in the VO_2_ and HR parameters, that would have been higher for the same RPE. This influence of PBC on RPE indicates a strong impact of cryostimulation on fatigue, also shown by the lack of significance shown by the VAS relative to muscle pain.

We must certainly highlight how the great variability of the measures is a factor to consider in the interpretation of the results. It is known that the coefficient of variation of the TTE is large. Despite constant-work test has a much lower coefficient of variation, with amateur athletes we must also consider the variability generated by pacing behavior. Hinckson and Hopkins [43], using the relationship between exercise duration and power output showed that the reliability of the equivalent mean power calculated from the constant-power test (0.6%) is even better than from the constant-work test (1.0%). In addition, Laursen and colleagues [44] reported that a small random variation in performance results in large variation in time to exhaustion, which causes large value of coefficient of variation. On the other hand, the small change after an intervention also leads to a large change in the time to exhaustion, therefore the signal-to-noise ratio of the constant-power test should be comparable to the constant-work test. In addition, participants were familiar with the experimental procedures, but especially in the TTE, the nature of the performance was far from the performance habits of the participants.

An important limitation of the present study is the lack of cardiovascular and physiological measures that compared the effects of the exposure to the PBC itself. Defining cardiovascular changes will be crucial for the assessment of the efficiency of PBC in sport.

Since the implementation of cryostimulation prior to competition is relatively recent [45], there is a lack of comparability among methodologies, in terms of cryostimulation modalities, exposure parameters, and types of exercise. Besides, future investigations should evaluate whether the exercise-induced inflammatory response is preserved in warmer conditions, and whether precooling effects are likewise effective with higher temperatures. Moreover, since we included amateur master athletes, future studies should aim to verify if the results of the present study could be extended to other population of athletes (e.g. young, elite, etc.). In addition, the 48-h TQR values could indicate an inadequate recovery for participants who underwent the subsequent visit after 48 h. However, most of them completed each visit with at least 72 h of recovery in between.

### Practical applications

This study results advocate for the use of cryostimulation before anerobic activities. Beyond the recovery benefits reported in the literature, cryostimulation may boost performance in the pre-execution phase. However, it is essential to evaluate the best timeframe to maximize the potential enhancing-performance effects. Cryostimulation may be also reducing the perception of effort and this effect could be crucial for coaches to modulate the training prescription of their athletes. Considering this result, athletes could potentially increase their external training load given the lower internal/perceptual load for the same external load. In fact, reducing RPE for the same external load could lead to a higher number of repetitions during an interval training session or a higher external intensity for each repetition.

## Conclusions

A single PBC session may represent a favorable set-up routine before running, improving middle-distance running performance and reducing RPE for the same external effort also in moderate-temperature conditions. Future studies should investigate the optimal integration of PBC with the traditional elements of active of pre-exercise routines, so to maximize middle-distance performance.

## Supporting information

S1 Data (XLSX)
